# Angiotensinogen and C3 compete for renin-induced complement activation

**DOI:** 10.3389/fimmu.2025.1563868

**Published:** 2025-04-02

**Authors:** Ann-Charlotte Kristoffersson, Albin Sköld, Charlotte Welinder, Markus Wendler, Gabriella Kalliokoski, Zivile Bekassy, Diana Karpman

**Affiliations:** ^1^ Department of Pediatrics, Clinical Sciences Lund, Lund University, Lund, Sweden; ^2^ Mass Spectrometry, Clinical Sciences Lund, Lund University, Lund, Sweden

**Keywords:** C3, complement, renin, angiotensinogen, kidney

## Abstract

Renin from plasma, kidney, and recombinant sources was previously demonstrated to cleave C3 to C3a and C3b. C3a was generated at a similar rate to that by C3 convertase, and C3 cleavage was inhibited by the renin inhibitor aliskiren. Renin endogenously produced by Calu6 cells also led to C3 deposition on cells. These results have been challenged by another group suggesting that recombinant renin does not cleave C3 or that renin was contaminated by trypsin, which also cleaves C3. Here, we investigated C3 cleavage by recombinant renin and competitive inhibition in the presence of angiotensinogen. Recombinant renin was analyzed by mass spectrometry using endopeptidase LysC digestion and did not contain trypsin. C3 cleavage, using our protocol and that of the other group, showed cleavage to C3b by immunoblotting. Cleavage was inhibited by aliskiren, which inhibits renin but not trypsin. Cleavage to C3a occurred within 1 min as detected by enzyme-linked immunosorbent assay (ELISA). Angiotensinogen competed for renin-mediated C3 cleavage and inhibited C3a generation, but C3 did not inhibit cleavage of angiotensinogen to angiotensin I (detected by ELISA). The results suggest that renin cleaves C3 but angiotensinogen is its preferred substrate. The interaction between renin and C3 may gain importance in the kidney where renin concentrations are considerably higher than in the circulation and when the primary substrate, angiotensinogen, is cleaved and thereby depleted.

## Introduction

Complement C3 cleavage is a pivotal step in the activation of the immune system, linking innate immune responses to downstream inflammatory processes. C3 is cleaved by the C3 convertase [C3(H_2_O)Bb and C3bBb], generating the anaphylatoxin C3a, and C3b, a central component of the C3 convertase. C3b binds complement factor B, cleaved by factor D to factor Ba and Bb, thereby forming more C3 convertase. In addition to C3 convertase, other enzymes are capable of cleaving C3 and generating C3a, such as kallikrein (1), plasmin, and thrombin (2, 3). In 2018, our group described renin-mediated C3 cleavage to C3a and C3b (4). The cleavage site was determined by N-terminal sequencing. Three purified sources of renin were used, plasma (5) (previously commercially available), kidney, and recombinant renin. C3 cleavage was demonstrated in purified form, as well as in serum, and was blocked by the renin inhibitor aliskiren or by pepstatin that inhibits aspartate proteases. Cleavage was demonstrated by the detection of C3b (by immunoblotting), C3a (by ELISA and immunoblotting), and by N-terminal sequencing of the C3b product. The C3b, generated by renin, bound factor B leading to the release of factor Ba, in the presence of factor D. The C3a generated led to mast cell chemotaxis. Complement activation was demonstrated on Calu6 cells that produce native renin, in the absence of exogenous renin, and C3 deposition on the cells was decreased by silencing of renin or treatment of the cells with aliskiren. Renin is released from juxtaglomerular cells in the kidney and aliskiren concentrations are considerably higher in the kidney than in the circulation (6). We therefore suggested that the effects of renin and its inhibitor aliskiren would be most pronounced in the kidney, especially in complement-mediated diseases, such as C3 glomerulopathy, and that systemic levels of C3 could still be low due to the presence of nephritic factors or complement gene variants (4). The effects of renin could not be tested *in vivo* in a mouse model as murine renin did not cleave murine C3 (4). We could demonstrate a beneficial effect of aliskiren administration in three C3 glomerulopathy patients treated for 4–7 years and on this basis initiated a clinical trial comparing aliskiren with enalapril in patients with C3 glomerulopathy (https://clinicaltrials.gov/study/NCT04183101).

Recently, another group, in Iowa, reported findings indicating that renin does not cleave C3 and suggesting that the renin used in our study could have been contaminated with trypsin (7). The plasma renin we previously used (4) (Biopur, Reinach Switzerland) was purified without trypsin (5). The Iowa group provided a list of recombinant renins and found trypsin contamination in two of these but the recombinant renin used in our previous study was not contaminated by trypsin (7). Here, we provide evidence that recombinant renin cleaves C3, using the protocols from Iowa and from Lund, and does not contain trypsin. Furthermore, we show that angiotensinogen, the known physiological substrate of renin (8), competitively inhibits C3 cleavage by renin.

## Methods

### Proteomics

#### Sample preparation

Renin from Abcam (ab135012), 1 µl (1 mg/ml), was diluted to a final volume of 100 µl with 100 mM ammonium bicarbonate and reduced with dithiothreitol to a final concentration of 10 mM, heated to 56°C for 30 min followed by alkylation with iodoacetamide to a final concentration of 20 mM for 30 min at rt in the dark. Digestion was performed by overnight incubation with 2 µl 0.1 µg endopeptidase LysC/µl (Wako Chemicals, Mass Spectrometry Grade 10AU, catalogue number: 129-02541) at 37°C and terminated by 10 µl 10% trifluoroacetic acid. Samples were placed in a Speed Vac until dry and resolved in 10 µl 2% acetonitrile (ACN) containing 0.1% trifluoroacetic acid.

#### LC-MS analysis

The mass spectrometry sample, 1 µl, was analyzed on an Exploris 480 mass spectrometer coupled to a Vanquish Neo UHPLC system (both from Thermo Fisher Scientific). Two-column setup was used on the HPLC system and peptides were loaded into an Acclaim PepMap 100 C18 precolumn (75 μm × 2 cm, Thermo Fisher Scientific, Waltham, MA) and then separated on an EASY spray column (75 μm × 25 cm, C18, 2 μm, 100 Å, ES902) with a flow rate of 300 nl/min. The column temperature was set at 45°C. Solvent A (0.1% formic acid, FA, in water) and solvent B (0.1% FA in 80% acetonitrile) were used to create a 30 min nonlinear gradient from 5% to 25% of solvent B for 25 min and increased to 28% for 3 min and then further increased to 30% for 2 min to elute the peptides.

#### Data-dependent acquisition

Samples were analyzed with a data-dependent acquisition (DDA) in positive mode. The full MS1 resolution was set to 120,000 at m/z 200 and the normalized automatic gain control (AGC) target was set to 300% with the maximum injection time of 45 ms. The full mass range was set to 375–1500 m/z. Precursors were isolated with the isolation window of 1.3 m/z and fragmented by higher energy collision dissociation (HCD) with the normalized collision energy of 30. MS2 was detected in the Orbitrap with the resolution of 30,000. The normalized AGC target and the maximum injection time were set to 100% and auto, respectively. The intensity threshold for precursor selection was set to 8.0e^3^ and 30s dynamic exclusion was applied.

Raw DDA data were analyzed using Proteome Discoverer™ 2.5 Software (Thermo Fisher Scientific). Peptide identification employed SEQUEST HT and Mascot against the UniProtKB human (UP000005640), bovine (UP000009136) and a contaminants fasta file from Max Planck Institute of Biochemistry, Martinsried Germany. The search parameters included static modification (cysteine carbamidomethylation) and dynamic modifications (N-terminal acetylation, oxidation of methionine). Precursor tolerance was set to 10 ppm, and fragment tolerance was set to 0.02 ppm. Up to two missed cleavages were allowed, and Percolator was employed for peptide validation at a maximum *q*-value of 0.05. Extracted peptides were used for identification. Only high-confident peptides were considered for protein identification when two or more unique peptides were detected. The mass spectrometry proteomics data have been deposited to the ProteomeXchange Consortium via the PRIDE (9) partner repository with the dataset identifier PXD053116.

### C3 cleavage to C3b and C3a by renin

Renin cleavage by C3 to C3b was investigated using two separate protocols. The first, termed Iowa protocol, is in accordance with Zhang et al. (7). Human C3 (250 µg/ml, 1.35 µM, Complement Technology, Tyler, TX) was incubated alone or with recombinant renin (130 µg/ml, 2.9 µM, Abcam catalogue 135012, Boston MA) for 24h at 37°C. Samples were diluted in Dulbecco’s phosphate-buffered saline (PBS) with or without Mg^2+^ (0.5 mM, as Mg was used in the Iowa protocol). The C3/renin ratio in µg/ml was 1.9. The second, termed Lund protocol, is in accordance with Bekassy et al. (4). C3 (100 µg/ml) was incubated alone or with recombinant renin (400 µg/ml) as above. The C3/renin ratio in µg/ml was 0.25. In separate experiments, renin was incubated with C3 for 30 min**–**3h at a ratio of 1:1 (100 μg/ml final concentration of both). Samples were run on a 4%–20%, Mini-PROTEAN TGX Gel (Bio-Rad, Hercules, CA) with 1× Tris/Glycine/SDS Buffer (Bio-Rad) under reduced conditions and transferred using the Trans-Blot Turbo Transfer Pack (Bio-Rad). After blocking in 1× casein (Vector Laboratories, Toronto, ON) the membrane was incubated with goat anti-human C3c (6.6 μg/ml final concentration, Sigma-Aldrich, St. Louis, MO). Following washing steps with PBS-T (Sigma-Aldrich), the membrane was incubated for 1h with rabbit anti-goat immunoglobulins/horseradish peroxidase (HRP, 500 ng/ml, Agilent Technologies, Denmark). The membrane was developed using the PierceTM ECL Plus Western Blotting Substrate (Thermo Fisher Scientific, Rockford, IL) and detection performed with the ChemiDocTM Touch Imaging System (Bio-Rad) and ImageLab (Bio-Rad).

C3 cleavage was also tested by generation of C3a. C3 (100 μg/ml) was incubated with recombinant renin (50 μg/ml) for 1–90 min at 37°C. Samples were diluted in Dulbecco’s PBS and analyzed for C3a by an ELISA kit (Quidel, San Diego, CA). Detection was performed using a Glomax Discovery System (Promega, Madison, WI) at 450 nm absorbance.

### C3 cleavage by renin in the presence of aliskiren

Renin cleavage (final concentration 100 µg/ml) by C3 (100 µg/ml) was also performed in the presence of the renin inhibitor aliskiren hemifumarate (final concentration 50 mM, Tocris Bioscience, Bristol, UK) diluted in 10× PBS. Renin was preincubated with aliskiren for 2h and after addition of C3 an additional 18h at 37°C. The C3 convertase C3bBb was formed by incubating C3 (100 μg/ml) with factor B (final concentration 50 μg/ml) and factor D (4 μg/ml, both from Complement Technology) in the presence of Ni2+ (Sigma-Aldrich). C3 cleavage to C3b was detected by immunoblotting as described above.

### C3 cleavage by trypsin in the presence of aliskiren

Porcine trypsin (final concentration 0.5 µg/ml, Thermo Fisher Scientific) was used to induce C3 cleavage (100 µg/ml) in the presence or absence of aliskiren (final concentration 0.04 M diluted in Dulbecco’s PBS, Capricorn Scientific, Ebsdorfergrund, Germany). Trypsin was preincubated with aliskiren for 30 min and after addition of C3 an additional 15 min at 37°C. C3 cleavage to C3b was detected by immunoblotting as described above.

### C3 cleavage by renin in the presence of angiotensinogen

#### C3a ELISA

C3 (200 μg/ml) was incubated alone, with recombinant renin (100 μg/ml) or with recombinant renin combined with human angiotensinogen (AGT) at increasing concentrations (62, 124, 248, and 496 μg/ml corresponding to 1, 2, 4, and 8 μM, respectively, Sigma-Aldrich) for 10 min at 37°C. Samples were analyzed for C3a by ELISA as described above.

#### C3b detection

Human C3 (400 μg/ml) was incubated alone, or with recombinant renin (100 μg/ml) or with recombinant renin and human AGT (248 μg/ml), for 6h at 37°C. C3 cleavage to C3b was detected by immunoblotting as described above. Band intensity of the detected α’ bands was calculated as a percentage of the corresponding β band.

### Angiotensinogen cleavage by renin in the presence of C3

#### Angiotensin I ELISA

An angiotensin (Ang) I ELISA kit (ALPCO Diagnostics, Salem, NH) was used to evaluate AGT cleavage to Ang I by renin in the presence or absence of increasing amounts of C3. AGT (124 µg/ml) was incubated alone, with renin (100 µg/ml) or with C3 (400, 800, 1200 and 1600 µg/ml). Detection was performed using the Glomax Discovery System.

### Statistics

Data were assessed by one-way analysis of variance and multiple group comparisons performed using the Games Howell *post-hoc* test or assessed by the Kruskal–Wallis test followed by Dunn’s multiple comparisons test. Assays were performed using R (CRAN, version 4.3.2) or GraphPad (version 9). A *P*-value ≤ 0.05 was considered significant.

## Result

### Recombinant renin does not contain trypsin

Mass spectrometry was used to analyze the content of recombinant renin and rule out the presence of trypsin. Recombinant renin from Abcam (catalogue number: Ab135012) did not contain any traces of trypsin, human UniProtKB (UP000005640), bovine (UP000009136), or porcine (contaminants fasta file from Max Planck Institute of Biochemistry, Munich-Martinsried, Germany). The mass spectrometry data are available via ProteomeXchange with identifier PXD053116.

### Evidence that recombinant renin cleaves C3 to C3b

**Figure 1:**
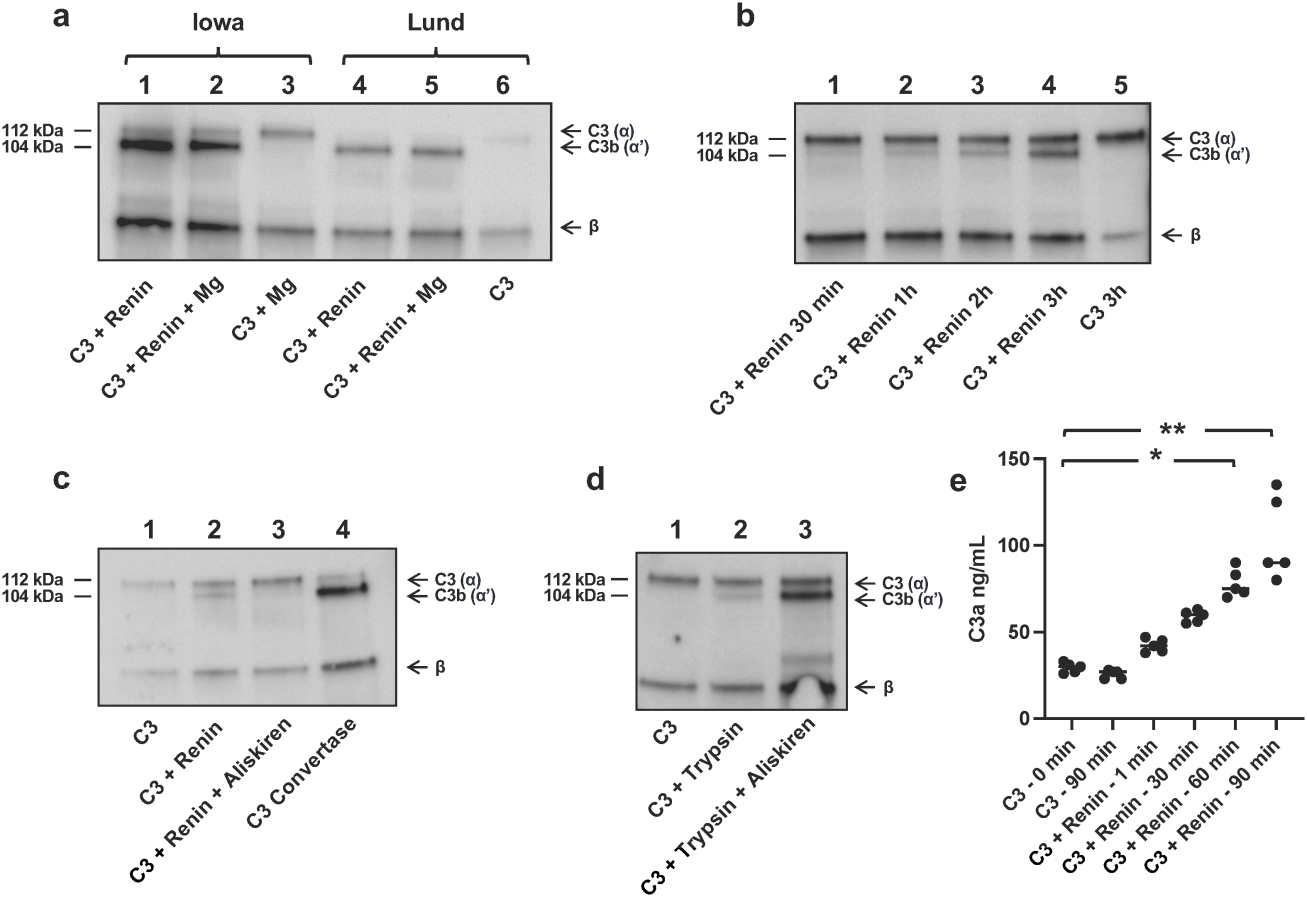
Renin cleavage of C3 is inhibited by aliskiren. **(a)** Renin-mediated C3 cleavage to C3b using the Iowa protocol (7) without magnesium (Mg, lane 1) and with Mg as per the reference (lane 2). C3 alone, as the negative control, using the Iowa protocol with Mg (lane 3). Total C3 cleavage to C3b using the Lund protocol (4) without Mg (lane 4) and with Mg (lane 5). C3 alone as per the Lund protocol (lane 6). All incubations were performed for 24h at 37°C. The figure shows one representative experiment, similar results were obtained in five separate experiments. **(b)** C3 incubated with renin (Lund protocol) for shorter incubation times of 30 min to 3h. **(c)** C3 alone showed no cleavage (lane 1). C3 incubated with renin showed cleavage to C3b (lane 2). C3 incubated with renin and aliskiren showed no cleavage (lane 3). Renin was preincubated with aliskiren for 2h and after addition of C3 an additional 18h at 37°C. For comparison, C3 convertase-mediated cleavage of C3 is shown in lane 4. **(d)** C3 alone showed no cleavage (lane 1), C3 incubated with trypsin (0.5 µg/ml) showed cleavage to C3b (lane 2), and C3 incubated with trypsin and aliskiren showed cleavage to C3b and lack of inhibition (lane 3). Trypsin was preincubated with aliskiren for 30 min and with C3 an additional 15 min at 37°C. All lanes were run on the same gel as shown and C3 detected by immunoblotting. Full blot images corresponding to panels a–d are presented in the supplement. **(e)** C3a generation when recombinant renin was incubated with C3 for up to 90 min. The two left columns show background C3a present in the sample before and after incubation for 90 min in the absence of renin. Statistical analysis was performed by Kruskal–Wallis test followed by Dunn’s multiple comparisons test. *P < 0.05, **P < 0.01.

A comparison was carried out between the Iowa protocol for cleavage of renin (7) with our own (Lund protocol). The Iowa protocol had a C3/renin ratio of 1.9 (in µg/ml) and the Lund protocol utilized a ratio of 0.25, that is, our protocol had more renin per C3. Both conditions cleaved C3 (**Figure 1a**, for the full-length blot, see **Supplementary Figure S1**). Cleavage was evident by a weak band corresponding to C3b by immunoblotting, detected after 30 min incubation and increasing in strength after 3h (**Figure 1b**, for the full-length blot see **Supplementary Figure S2**). C3 cleavage by renin was inhibited by aliskiren (**Figure 1c**, for the full-length blot see **Supplementary Figure S3**). Trypsin also cleaves C3, but trypsin-mediated C3 cleavage was not inhibited by aliskiren (**Figure 1d**, for the full-length blot see **Supplementary Figure S2**), further indicating that the C3 cleavage induced by renin could not be mediated by trypsin contamination. Taken together, these lines of evidence demonstrate that recombinant renin cleaves C3 and is not contaminated by trypsin.

### Evidence that recombinant renin cleaves C3 to C3a

Incubation of renin with C3 yielded C3a, which was demonstrated within 1 min and further increased during a 90-min incubation (**Figure 1e**).

### Angiotensinogen inhibits renin-mediated C3 cleavage

**Figure 2:**
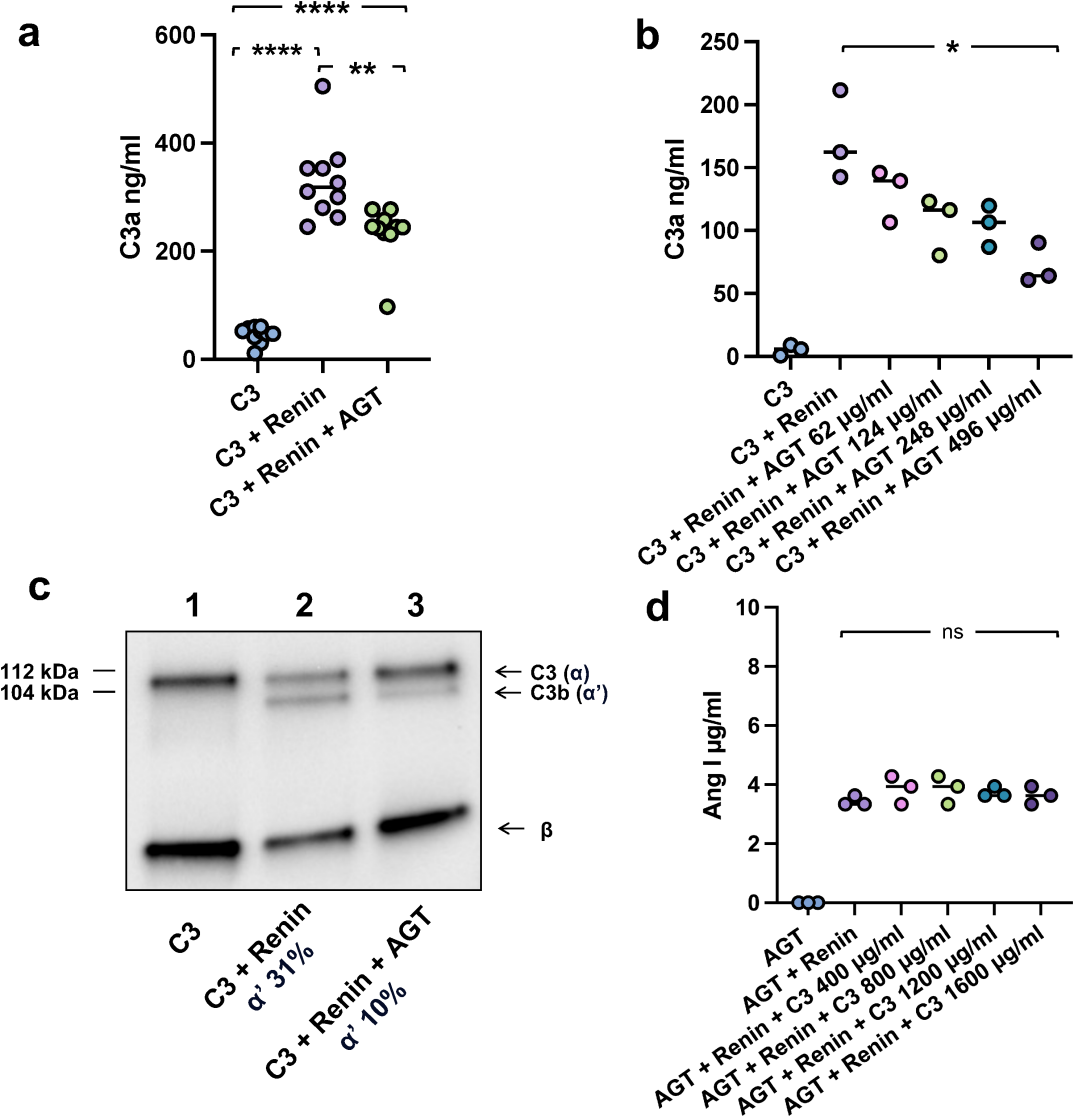
Angiotensinogen competes with C3 as a substrate of renin. **(a)** C3a generation following C3 incubation alone (400 µg/ml), C3 with renin or C3 with renin and angiotensinogen (AGT 124 µg/ml) for 10 min at 37°C. Renin-mediated C3 cleavage to C3a was partly inhibited in the presence of angiotensinogen. The bar denotes the median. **(b)** C3a generation as in panel a following incubation of C3 alone (200 µg/ml) with varying concentrations of AGT showing a dose-dependent decrease in C3a generation. **(c)** C3b generation when C3 (400 µg/ml) was incubated alone (lane 1), C3 with renin 100 µg/ml (lane 2), or C3 with renin and AGT 248 µg/ml (lane 3) for 6h at 37°C. C3 was cleaved to C3b by renin. Band intensity of the detected α’ bands were calculated as a percentage of the corresponding β band. C3 cleavage was inhibited in the presence of angiotensinogen. Detection performed by immunoblot. **(d)** Angiotensin I (Ang I) generation following renin incubation with angiotensinogen with or without C3 at varying concentrations at 37°C. C3 had no effect on renin-mediated cleavage of angiotensinogen to angiotensin I Statistical analysis performed by Games Howell multiple comparisons (panel a) and Kruskal–Wallis test followed by Dunn’s multiple comparisons test (panels b and d). *P < 0.05, **P < 0.01, ****P < 0.0001. ns, not significant. Full blot image corresponding to **(c)** is presented in the supplement.

As renin cleaves both C3 and angiotensinogen these substrates could compete for interaction with the enzyme. When co-incubating both substrates with renin, angiotensinogen inhibits C3 cleavage and generation of C3a (**Figures 2a, b**) as well as C3b (**Figure 2c**), demonstrating that there is competitive inhibition between the two substrates. As shown in **Figure 2b**, the highest concentration of angiotensinogen (8 μM) inhibited 60% of C3 cleavage and the inhibitory pattern was linear. While angiotensinogen interferes with C3 cleavage the converse could not be shown, that is, C3 does not inhibit the cleavage of angiotensinogen to angiotensin I (**Figure 2d**), suggesting that renin prefers angiotensinogen over C3 in the fluid phase. Thus, the competitive inhibition between the two renin substrates in the circulation, and renin’s preference for angiotensinogen, should be considered when assessing renin-mediated C3 cleavage.

## Discussion

This study demonstrates that renin cleaves C3 and the interaction is inhibited by the renin inhibitor aliskiren, and by the alternative substrate, angiotensinogen, which was shown to be the preferred substrate. Rapid renin-mediated C3a generation suggests that the interaction may occur when levels of angiotensinogen are low, such as when the substrate is depleted (10) and cleaved to angiotensin I. The interaction of renin with C3 will gain importance in the kidney where levels of renin are higher than in the circulation as renin is solely released in the kidney (11).

On Calu6 cell surfaces we previously showed C3 deposition related to the presence of renin but without adding exogenous enzyme (4). Using MES-13 cells Zhang et al. could not demonstrate C3 deposition in the presence of exogenously added renin (7). This discrepancy could be related to very different experimental conditions used, distinctively different cells, and importantly, in our experiments renin was not added, rather native renin was released by the cells themselves and the effect blocked by silencing or by coincubation with aliskiren.

The interaction between angiotensinogen, C3, and renin suggests that the renin-angiotensin and complement systems can interact in the circulation and angiotensinogen inhibits C3 cleavage by renin. Therefore, levels of serum renin should not necessarily correlate to C3 levels as renin would mostly be occupied by angiotensinogen cleavage in the systemic circulation. Likewise, patients with complement-mediated kidney disease, such as C3 glomerulopathy, are often treated with angiotensin-converting enzyme (ACE) inhibitors or angiotensin receptor blockers, which markedly increase renin levels. Thus, renin/C3 correlations would be skewed due to pharmacological intervention thereby increasing renin, and the presence of nephritic factors or genetic variants in C3 glomerulopathy (12), which would lower C3 levels. This aspect could lead to misinterpretation of actual renin/C3 ratios in the circulation.

Molecular modelling was used to investigate the interaction between renin and its two substrates, angiotensinogen and C3 (7). In both cases a 14-amino acid peptide was used instead of the entire protein. The peptide-based approach may be insufficient for the study of the binding sites in renin. Structural studies of the interaction between renin and angiotensinogen have demonstrated that the tertiary structure of angiotensinogen is of crucial importance for binding (8). Angiotensinogen undergoes a profound conformational change during this process and release of angiotensin I from angiotensinogen is higher for the entire protein compared to the 14-amino acid peptide (8). Likewise, using a 14-amino acid peptide for determining the interaction between renin and C3 may be inappropriate. However, even if the use of the entire protein had been carried out and showed that renin binds angiotensinogen preferentially that would be in line with the findings outlined herein and would not rule out the capacity of renin to cleave C3 under certain conditions, which we show unequivocally.

**Figure 3:**
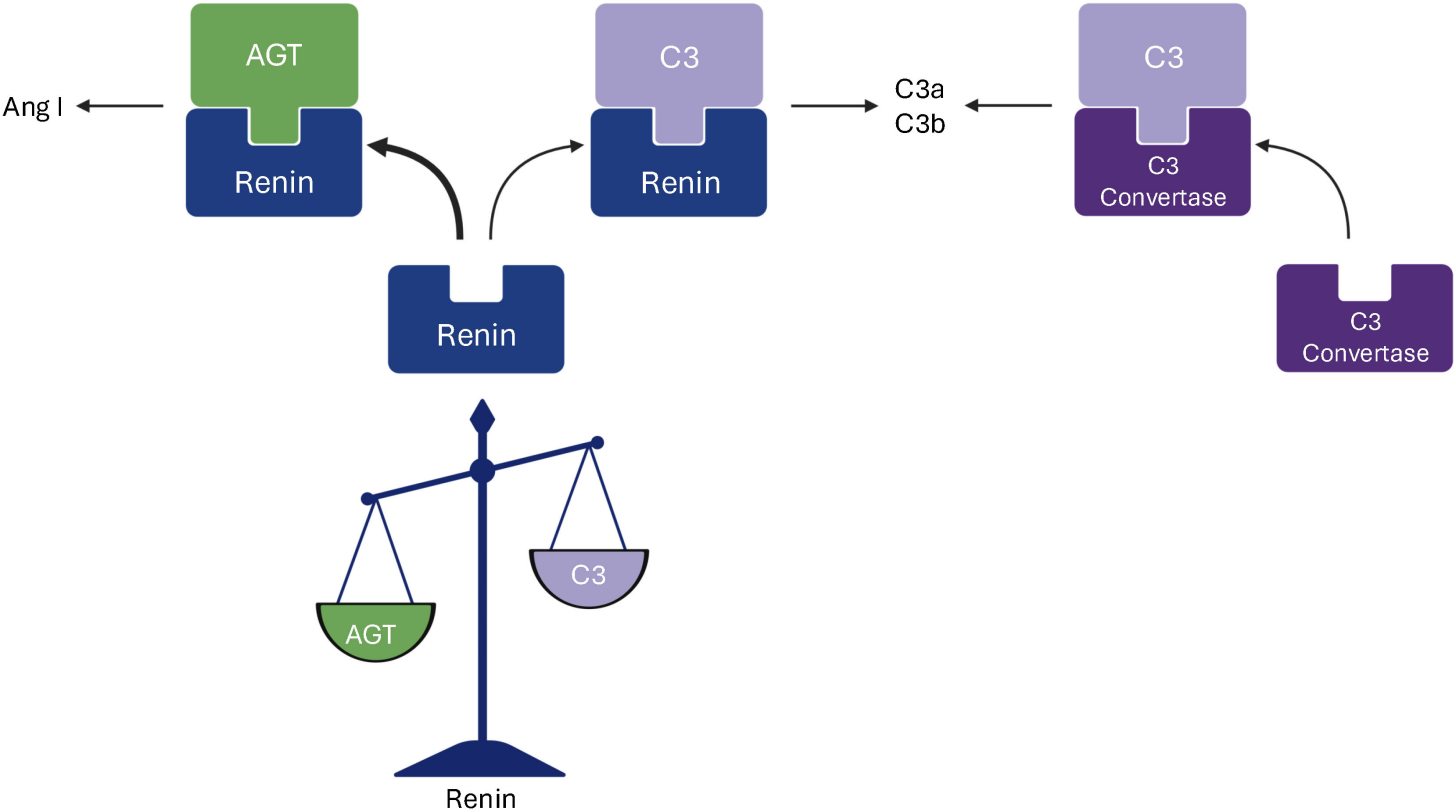
A schematic presentation of renin-mediated cleavage of C3. This study demonstrates that renin cleaves both angiotensinogen and C3 and that angiotensinogen is its preferred substrate, resulting in cleavage to angiotensin I (Ang I). C3 is cleaved to C3a and C3b by both the C3 convertase and renin. Figure created with Biorender.

The evidence in this study strongly supports our previous study showing that renin cleaves C3, *in vivo* this would presumably occur predominantly on cell surfaces in the kidney where concentrations are higher. Additionally, angiotensinogen competes with C3 for renin binding and enzymatic activity, particularly in the circulation, and aliskiren inhibits complement activation induced by renin. The results are schematically presented in **Figure 3**. We deduce that renin-mediated C3 cleavage has a role in complement activation, particularly in the kidney. The results suggest that renin may act as a novel modulator of the complement system, particularly in the kidney, where local inflammation could amplify immune responses in diseases such as lupus nephritis or C3 glomerulopathy.

## Data Availability

The datasets presented in this study can be found in online repositories. The names of the repository/repositories and accession number(s) can be found below: PXD053116 (PRIDE).
