# Supplementary material for: Angiotensinogen and C3 compete for renin-induced complement activation
Source: Front Immunol. 2025 Apr 2;16:1563868. doi: 10.3389/fimmu.2025.1563868 (PMC11999838; doi:10.3389/fimmu.2025.1563868)
Supplement: Supplementary file 1 [file DataSheet1.pdf]

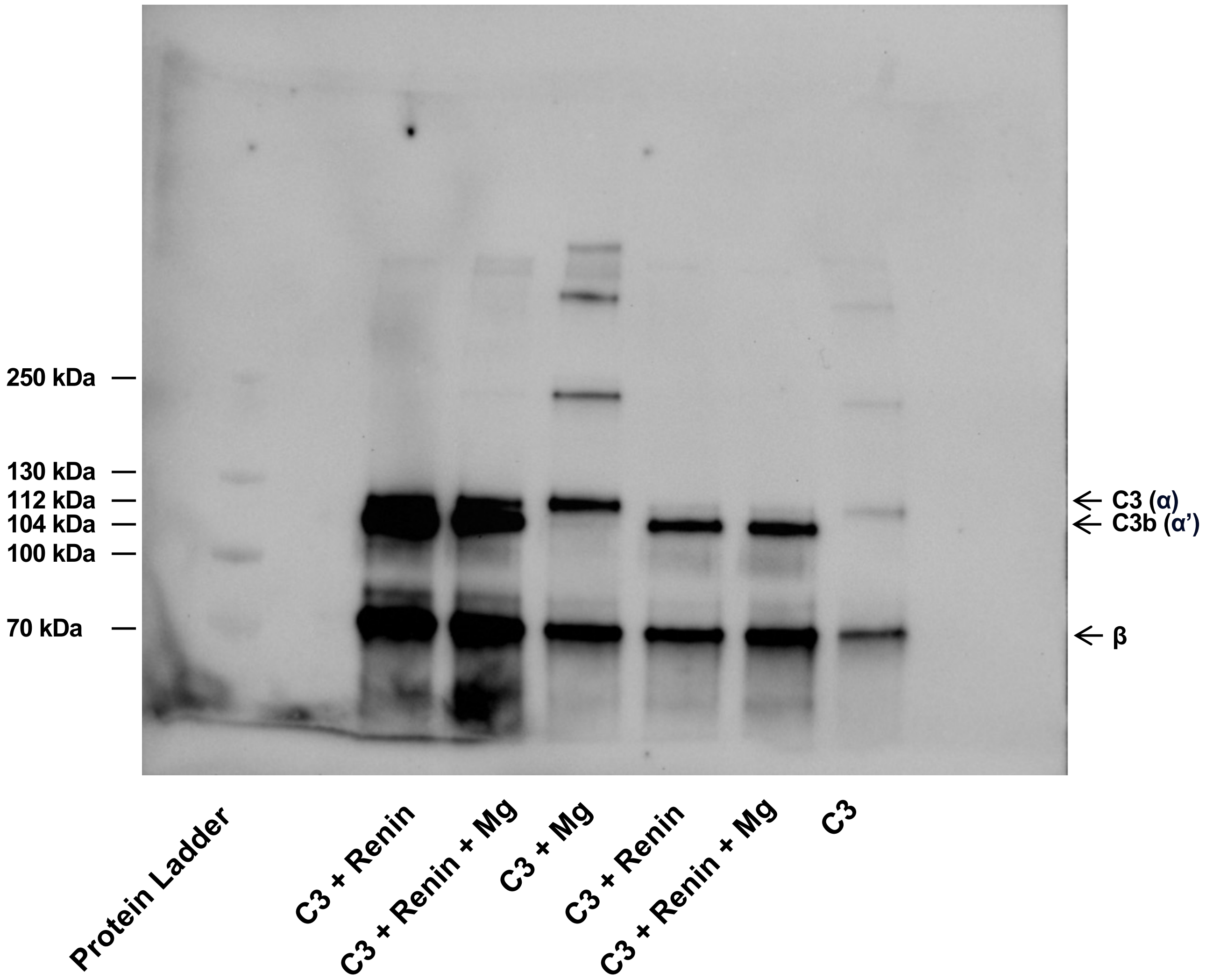

Supplementary Figure 1: Panel 1a full blot

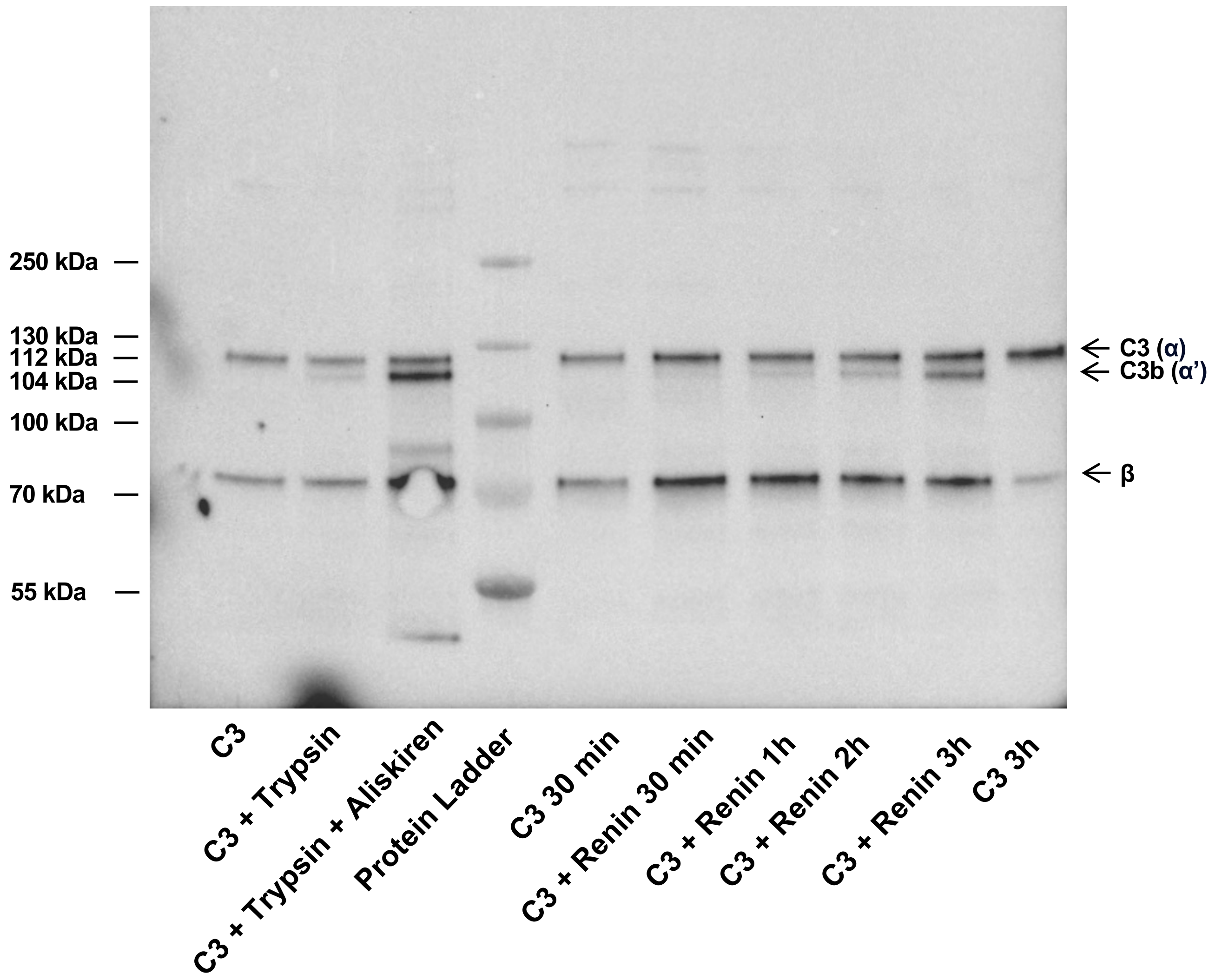

Supplementary Figure 2: Panel 1d and 1b full blot

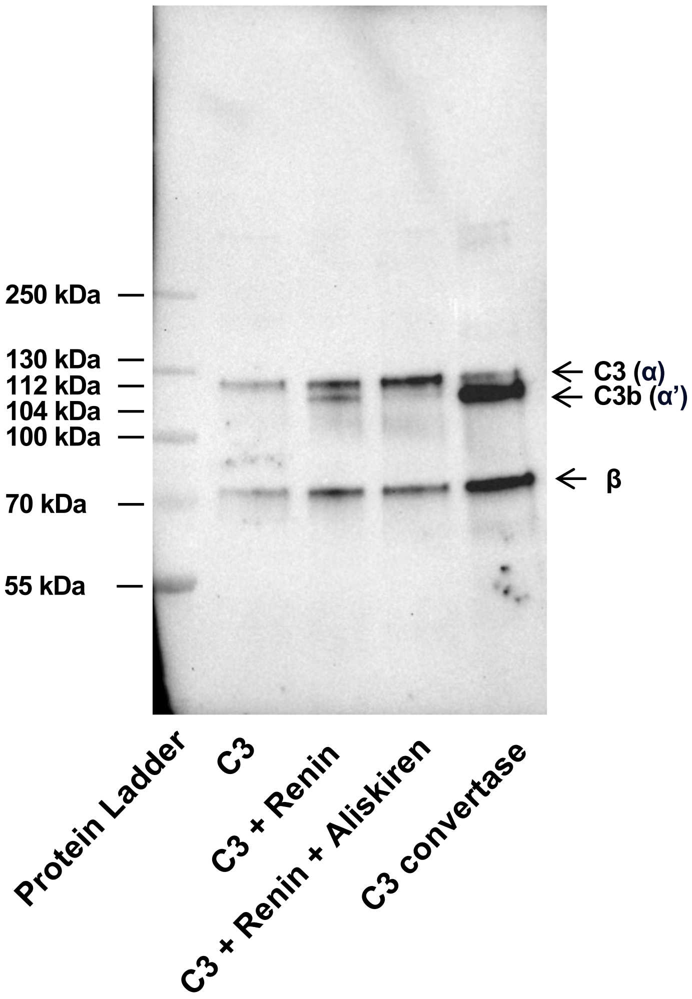

Supplementary Figure 3: Panel 1c full blot
